# Longitudinal Pathogenic Properties and *N*-Glycosylation Profile of Antibodies from Patients with Pemphigus after Corticosteroid Treatment

**DOI:** 10.3390/biomedicines9101411

**Published:** 2021-10-08

**Authors:** Marie Petit, Marie-Laure Walet-Balieu, Damien Schapman, Marie-Laure Golinski, Carole Burel, Marion Barray, Laurent Drouot, Maud Maho-Vaillant, Vivien Hébert, Olivier Boyer, Muriel Bardor, Pascal Joly, Sébastien Calbo

**Affiliations:** 1 INSERM U1234, Normandie University, 76000 Rouen, France; marie.petit1994@gmail.com (M.P.); marie-laure.golinski@chu-rouen.fr (M.-L.G.); marion.barray@hotmail.com (M.B.); Laurent.Drouot@univ-rouen.fr (L.D.); maud.maho@univ-rouen.fr (M.M.-V.); vivien.hebert@chu-rouen.fr (V.H.); olivier.boyer@chu-rouen.fr (O.B.); Pascal.Joly@chu-rouen.fr (P.J.); 2 EA4358, Laboratoire Glycobiologie et Matrice Extracellulaire Végétale (Glyco-MEV), Normandie University, 76821 Rouen, France; marie-laure.walet-balieu@univ-rouen.fr (M.-L.W.-B.); carole.burel@univ-rouen.fr (C.B.); muriel.bardor@univ-rouen.fr (M.B.); 3 PRIMACEN, Normandie University, 76821 Rouen, France; damien.schapman@univ-rouen.fr; 4 Dermatology Department, Rouen University Hospital, Normandie University, 76000 Rouen, France; 5 CNRS UMR 8576, Unité de Glycobiologie Structurale et Fonctionnelle, University of Lille, 59000 Lille, France

**Keywords:** autoantibodies, pemphigus, pathogenic, glycans

## Abstract

Pemphigus vulgaris is an autoimmune disease that occurs due to pathogenic autoantibodies that recognize the following epidermal adhesion proteins: desmogleins. Systemic corticosteroids usually decrease the titers of anti-desmoglein autoantibodies and improve patients’ conditions. Since modifications of IgG *N*-glycosylation have been described in some autoimmune diseases, we hypothesized that changes in the pathogenic activity of pemphigus IgG could be related to changes in their *N*-glycosylation profile. The purpose of this study was to assess, longitudinally, the pathogenicity of pemphigus serum IgG and their *N*-glycosylation profile during phases of disease activity and clinical remission. The pathogenic activity of serum IgG was measured in vitro on immortalized keratinocytes, by immunofluorescence and dissociation assays, and IgG *N*-glycans were analyzed by mass spectrometry. We showed (i) a correlation between pemphigus clinical activity and the pathogenicity of serum IgG at baseline and at month 6, while the persistence of the in vitro pathogenic activity of IgG during its evolution, even in patients in clinical remission, seemed to be predictive of relapse; (ii) that modifications of the *N*-glycan structure were altered the in vitro pathogenicity of patients’ autoantibodies; (iii) that the pathogenic properties of pemphigus IgG did not appear to be related to the disparity in IgG *N*-glycans during the course of pemphigus.

## 1. Introduction

Pemphigus vulgaris (PV) is a rare life-threatening autoimmune blistering disease of the skin and mucous membranes, with an estimated 0.6 (in Switzerland) to 10 (in Iran) individuals per 1 million people per year being affected worldwide [1]. PV is characterized by the production of pathogenic autoantibodies targeting desmoglein (DSG) 1 and 3, which are transmembrane glycoproteins of desmosomes that confer cell–cell adhesion within the epidermis [1]. The pathogenicity of circulating IgG antibodies from pemphigus patients has been confirmed by passive transfer experiments in mice [2,3]. The observation of neonatal PV in newborns of mothers with PV corresponding to a passive transfer in humans, confirmed that autoantibodies are sufficient to cause the disease [4,5]. Mechanistically, autoantibodies interfere with cell adhesion by steric hindrance of DSG adhesion, or induce DSG internalization either by autoantibody clustering or by cell signaling (mainly Src and p38 MAPK), which contribute to the loss of cell adhesion [6].

Among IgG isotypes, IgG4 is the least represented in the serum of normal individuals, corresponding to 5% of the total IgG [7]. Additionally, IgG4 is not able to activate complement via the classical pathway [8]. In contrast, there is a predominance of the IgG4 subclass in the serum of pemphigus patients, both in the DSG-specific IgG and among the total IgG [9,10]. IgG1 anti-DSG Ab are also frequently detected, while IgG2 and IgG3 anti-DSG Ab are less frequently detected in patients with active pemphigus [11]. In patients in clinical remission, as in healthy relatives, anti-DSG IgG2 is the most frequently detected IgG subclass, and is sometimes associated with IgG1 or IgG4, while the combination of IgG1 and IgG4 anti-DSG Ab was not observed in any case [12]. Interestingly, altered *N*-glycosylation of IgG4 can activate complement via the lectin pathway in membranous nephropathy [13], as well as in rheumatoid arthritis (RA) [14].

IgG isotypes have a conserved *N*-linked glycosylation site located at Asn297 on the CH2 domain of the Fc region. The *N*-glycans located at this site contribute to the formation of a hydrophobic pocket on the Fc domain, which stabilizes the immunoglobulin structure [15]. The composition of IgG *N*-glycans varies according to the IgG isotype [16]. In particular, variations in terminal sialic acid substitution, galactose, bisecting *N*-acetylglucosamine, and fucosylation of the core have been reported [16]. These modifications of the *N*-glycan structures can affect and modulate antibody functions, such as the binding to Fc receptors on effector cells [17]. In addition to the conserved IgG Fc *N*-glycans, 15 to 25% of serum IgGs contain *N*-glycans within their variable domain. These so-called “Fab *N*-glycans” mainly consist of highly processed, complex-type biantennary *N*-glycans linked to *N*-glycosylation sites that emerge during somatic hypermutation [18]. The amounts and types of *N*-glycans linked to either the Fab or Fc parts of the IgG can vary during specific physiological and pathological conditions. Notably, it has been reported that alterations in IgG and autoantibody *N*-glycosylation were detected in many autoimmune diseases [19]; for instance, a decreased level of IgG galactosylation has been observed in systemic lupus erythematosus (SLE), inflammatory bowel disease, and anti-neutrophil cytoplasmic antibody-associated vasculitis [20,21,22]. On the contrary, a high level of IgG galactosylation and sialylation can induce anti-inflammatory activity, which might explain the persistence of circulating autoantibodies in some patients who are in clinical remission [19].

We previously demonstrated that around half of the PV patients treated with corticosteroids and/or immunosuppressants achieve complete remission off therapy [23,24]. Nevertheless, some of these patients in complete remission still had detectable anti-DSG antibodies [25]. In the present study, we longitudinally analyzed IgG from PV patients at four different time points, in order to correlate the clinical status of the patients, the in vitro pathogenic activity of their serum IgG, and their *N*-glycans profile.

## 2. Materials and Methods

### 2.1. Cell Culture

The human immortalized keratinocyte cell line HaCaT was cultured in Dulbecco’s modified Eagle medium (DMEM) GlutaMAX, high glucose (Thermo Fisher Scientific, Waltham, MA, USA) containing 10% FCS (fetal calf serum, Eurobio Scientific, Les Ulis, France), 50 U/mL penicillin and streptomycin (Thermo Fisher Scientific, Waltham, MA, USA) in a humidified atmosphere of 5% CO_2_ at 37 °C.

### 2.2. Sera and IgG Purification

Pemphigus sera were collected throughout a randomized clinical trial (RITUX 3: ClinicalTrials.gov number, NCT00784589, accessed on 6 August 2021); patients were treated with oral prednisone alone or a combination of rituximab and short-term prednisone regimen [24]. Anti-DSG1 and anti-DSG3 IgG antibody titers were measured by ELISA (Euroimmun, Lübeck, Germany). The mouse monoclonal antibody (mAb) AK23 was purified from AK23 hybridoma culture supernatant, kindly provided by Dr Masayuki Amagai (Keio University, Minato City, Tokyo, Japan). AK23 is an anti-DSG3 mAb generated in a PV mouse model that is pathogenic both in vitro and in vivo [26,27]. AK23 mAb or serum IgG from anti-DSG3^+^ patients or from healthy donors (HD) were purified by automated protein-G affinity purification on an ÄKTA Start chromatograph using HiTrap^®^ Protein G High Performance (GE Healthcare Chicago, IL, USA ) according to the manufacturer’s instructions. 

### 2.3. Antibodies

The following antibodies were used in immunofluorescence assays: anti-DSG3 Alexa Fluor 647 (clone: 5H10; Santa Cruz, Dallas, TX, USA), anti-flotillin-2 (Human Protein Atlas number: HPA001396; Sigma, Saint-Louis, MO, USA ) and then revealed by the secondary antibody Alexa Fluor 488 (Abcam, Cambridge, UK). 

### 2.4. In Vitro Modification of the N-Glycans of Purified IgG

For desialylation, 10 µg of AK23 mAb was incubated with 52.5 mU of neuraminidase from *Vibrio cholerae* (Sigma) for 120 h at 37 °C in a 50 mM sodium acetate buffer (pH 5). Enzyme and buffer were removed using Amicon Ultra 0.5 mL centrifugal filters with cut-off of 100 kDa (Merck Millipore, Burlington, MA, USA). Fifty micrograms of patient’s IgG was incubated with 37.5 mU for 120 h at 37 °C in a 50 mM sodium acetate buffer (pH 5). Enzymatic digestion efficiency was tested by Eastern blot using biotinylated conjugated *Maackia amurensis* lectin II (MAL II, Bioworld, Dublin, OH, USA) or *Sambucus nigra* lectin (SNA, EBL, Vector Laboratories, Inc, Burlingame, CA, USA). 

For degalactosylation, 10 µg of AK23 mAb was incubated with 28 mU of β-galactosidase from bovine testes (Sigma) for 72 h at 37 °C in a 50 mM sodium acetate buffer (pH 5) (complete digestion). Enzyme and buffer were then removed using Amicon Ultra 0.5 mL centrifugal filters with cut-off of 100 kDa (Merck Milipore, Burlington, MA, USA). Enzymatic digestion efficiency was tested by Eastern blot using biotinylated conjugated *Erythrina cristagalli* lectin (ECA, Vector Laboratories, Inc, Burlingame, CA, USA).

### 2.5. Eastern Blot

IgG was loaded on a 4–12% SDS-PAGE gel under reducing conditions, transferred to nitrocellulose or PVDF membrane using the iBlot 2 Dry Blotting System (Thermo Fisher) and blocked with PBS—0.1% bovine serum albumin (BSA). Blots were incubated with biotinylated conjugated *Maackia amurensis* lectin II (0.4 µg/mL; MAL II, Bioworld, Dublin, OH, USA) or *Sambucus nigra* lectin (SNA/EBL, Vector Laboratories, Inc, Burlingame, CA, USA) for sialic acid recognition or biotinylated conjugated *Erythrina cristagalli* lectin (1 µg/mL; ECA, Vector Laboratories, Inc, Burlingame, CA, USA) for galactose recognition, followed by incubation with streptavidin–protein, DyLight 800 (Thermo Fisher Scientific, Waltham, MA, USA) and detection using an Odyssey scanner (LI-COR Biotechnology LI-COR, Lincoln, NE, USA ). *Sambucus nigra* lectin binds preferentially to sialic acid attached to terminal galactose in α-2,6 while *Maackia amurensis* II appears to bind sialic acid in an α-2,3 linkage.

### 2.6. Dispase-Based Dissociation Assay

HaCaT cells were seeded onto 24-well plates and cultured in DMEM GlutaMAX medium containing 1 mM CaCl_2_ and 10% FCS to confluence. Subsequently, cells were incubated for 24 h at 37 °C with 10 µg/mL purified AK23 mAb or 62.5 µg/mL purified IgG from PV patients or HD. Cells were then washed with Hanks’ buffered saline solution (HBSS) 1× (GE Healthcare, Chicago, IL, USA) complemented with 3 mM of CaCl_2_ and incubated for 25 min with Dispase II (2.4 U/mL, Sigma) at 37 °C to detach them from the bottom of the plate. After washing with HBSS 1× complemented with 3 mM of CaCl_2_ and 5% FCS, the cells were stained with crystal violet (Sigma) and a mechanical stress was applied by pipetting the cells 5–10 times with a P1000 pipette. The plates were centrifuged at 300× *g* at room temperature (RT) for 5 min and the number of cell fragments was counted. A picture of each well was taken with EVOS XL core (Invitrogen, Waltham, MA, USA) microscope ×2 magnification. For this dissociation assay, we systematically used AK23 mAb and IgG from HD as positive and negative controls, respectively. Variation in the total number of fragments obtained with AK23 mAb or IgG from HD was observed from one experiment to another. In order to quantify the pathogenicity of IgG, we developed a score of pathogenicity based on the number of fragments obtained. To create this score, we used the number of fragments obtained after mechanical stress of the layer of keratinocytes incubated with HD IgG and the one obtained after incubation with AK23 mAb or with patient IgG, whichever fragmented the most, as low and high limit of the score, respectively. We used AK23 mAb as the high limit for experiments with only AK23 mAb, but due to its high pathogenicity, we used patient IgG as the high limit for the other experiments. In our score bar, 0 corresponded to the number of fragments below or equal to those obtained after incubation with IgG from HD; a score of 1 corresponded to the number of fragments between the number of fragments obtained after incubation with IgG from HD and 1/5 of the number of fragments obtained after incubation with AK23 mAb/patient IgG. Scores of 2, 3 and 4 corresponded to the number of fragments between 1/5 and 2/5, 2/5 and 3/5, 3/5 and 4/5 of the number of fragments obtained after incubation with AK23 mAb/patient IgG, respectively. Finally, a score of 5 corresponded to the number of fragments greater than 4/5 of the number of fragments obtained after incubation with AK23 mAb/patient IgG.

### 2.7. Immunofluorescence Assays

HaCaT cell line was seeded on glass coverslips with cell culture chambers (Nun Lab-Tek II Chamber Slide System, Thermo Fisher Scientific, Waltham, MA, USA) and cultured for at least 2 days in DMEM GlutaMAX medium containing 1 mM CaCl_2_ and 10% FCS and grown to confluence. Cells were treated with either IgG from PV patients’ or HD serum for 20 h in DMEM GlutaMAX medium containing 1 mM CaCl_2_ without FCS. After removing the medium and washing with PBS 1× complemented with CaCl_2_ and MgCl_2_ (Eurobio Scientific, Les Ulis, France), the cells were then fixed with ethanol 100% for 10 min at RT. The fixed cells were rinsed and permeabilized with Triton X-100 at 0.3% for 10 min (Sigma). After washing, cells were blocked for 30 min with 1% rat serum in PBS 1× at RT. Fluorescent-labeled antibodies staining DSG3 and flotillin-2, a protein associated with lipid microdomains that interact with desmosome proteins [28], were incubated for 1.5 h in the dark at RT in PBS 1× containing 1% BSA. Finally, coverslips were mounted with ProLong^™^ Diamond Antifade Mountant containing DAPI (Thermo Fisher Scientific, Waltham, MA, USA). All samples were analyzed with a Leica SP8-UV confocal microscope.

### 2.8. Mass Spectrometry Analysis of IgG N-Glycans

First, purified IgGs were deglycosylated by peptide *N*-glycosidase F (PNGase F, Roche, Bâle, Switzerland) digestion as previously reported [29]. Briefly, for PNGase F digestion, 0.5 mg of IgG was dissolved in 450 µL of (NH_4_)_2_CO_3_ (20 mM) and 50 µL of denaturing buffer containing 0.2% SDS and 100 mM of β-mercaptoethanol. The samples were heated for 10 min at 100 °C for protein denaturation. After cooling down, the digestion was performed with 10 units of PNGase F for 24 h at 37 °C. Finally, deglycosylated IgGs were precipitated by the addition of 4 volumes of ethanol overnight at −20 °C. After precipitation, the samples were centrifugated for 15 min at 4 °C at 11,400× *g* and the supernatant containing the released *N*-glycans was retrieved and placed in glass tubes for evaporation using an evaporator under gas flow. Then, the free *N*-glycans were permethylated as previously described [30]. The permethylated *N*-glycans were purified using C18 columns (Hypersep^™^ C18 Cartridges, 200 mg, 3 mL, Thermo Fisher Scientific, Waltham, MA, USA). First, columns were conditioned by successive washing with 5 mL of methanol, 5 mL of water, 5 mL of acetonitrile and 5 mL of water. Permethylated samples were resuspended in 200 µL of a solution of methanol/water 50/50 (*v*/*v*) and were passed over the column. Successive elution with 2 mL of 15% (*v*/*v*), 35% (*v*/*v*), 50% (*v*/*v*) and 75% of acetonitrile (*v*/*v*) were performed. Permethylated *N*-glycans were recovered in the 50% and 75% acetonitrile fractions. The resulting samples were dried in a Thermo Fisher SPD111 V SpeedVac^®^ prior to analysis using matrix-assisted laser desorption/ionization—time-of-flight mass spectrometry (MALDI-TOF MS; UltrafleXtreme time-of-flight mass spectrometer (Bruker Daltonics, Bremen, Germany) equipped with an Nd: YAG laser (355 nm wavelength), 2kHz smartbeam^TM^-II [31]). 

Samples were prepared for analysis as a mixture with dihydroxybenzoic acid (DHB) used as matrix. This matrix was freshly dissolved in 20 mg/mL of an 80% methanol solution. Permethylated *N*-glycans were solubilized in an acetonitrile/0.1% trifluoroacetic acid 70/30 *v*/*v*. Samples and matrix were spotted in ratio 1/1 *v*/*v*. These spectra were recorded in reflectron positive mode with an accumulation of minima 10,000 shots with FlexControl 3.4 software. Mass spectra were acquired using a mass range *m*/*z* 900–4500 and analyzed with FlexAnalysis 3.4 software (Bruker Daltonics, Bremen, Germany). Based on the *m*/*z* ratio and report from the literature on IgG *N*-glycosylation, each ion was associated with an *N*-glycan structure, which were drawn according to the international nomenclature recently updated [32]. 

To calculate the relative percentage of *N*-glycan intensity, the averages of particular *N*-glycosylation features (galactosylation, fucosylation, sialylation, additional *N*-acetylglucosamine) have been made from different individual *N*-glycan structures and their percentages have been calculated using the sum of the intensity of all the *N*-glycan species in the sample as 100%.

### 2.9. Statistical Analysis

All statistical analyses were performed using GraphPad Prism (GraphPad Software, La Jolla, CA, USA), using an unpaired, non-parametric, Mann–Whitney test. Differences were considered significant when *p* < 0.05.

## 3. Results

### 3.1. Role of N-Glycans in IgG Pathogenic Activity

**Figure 1:**
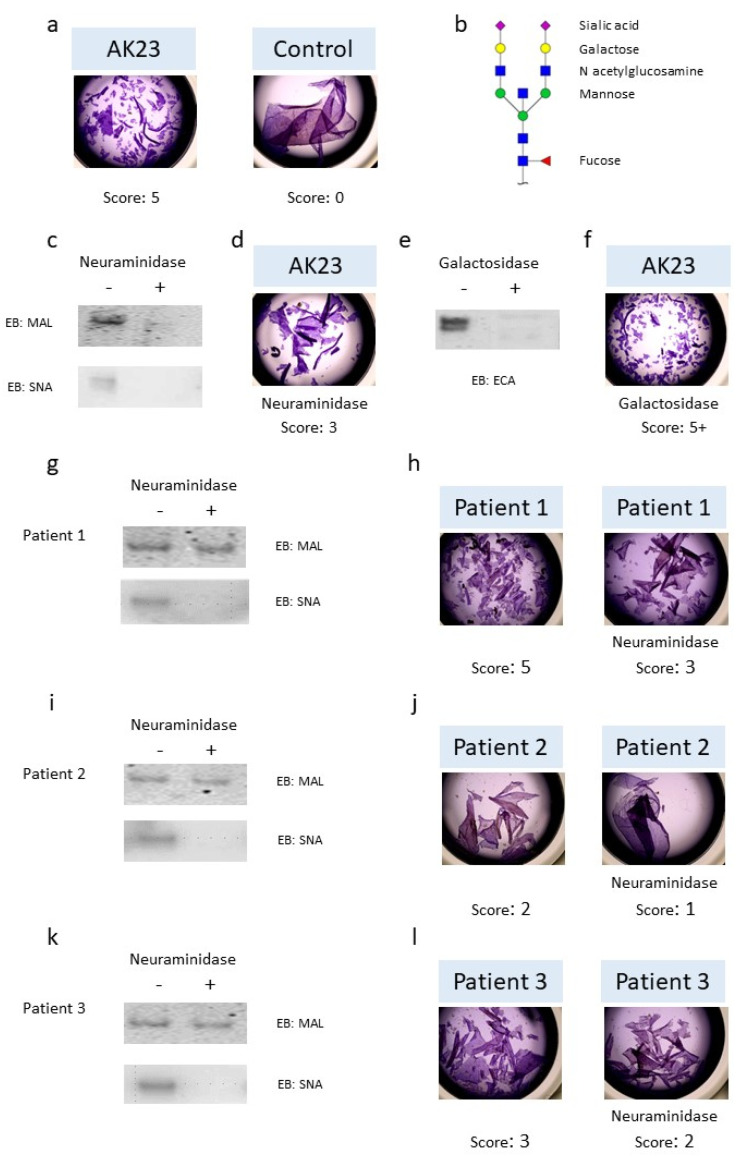
Pathogenicity of monoclonal antibody AK23 and pemphigus patients’ IgG collected during the active phase after the removal of sialic acid and galactose. (**a**) Monolayers of HaCaT cells were treated with monoclonal antibody AK23 (10 µg/mL) or with purified IgG (62.5 µg/mL) from healthy donors for 24 h. Dispase-based dissociation assay and calculation of the score were performed as described in the Section 2.6. (**b**) Typical sialylated *N*-glycan structure on IgG bearing a bisecting *N*-acetylglucosamine residue. (**c**) AK23 were treated by neuraminidase (+) or not (−) to remove sialic acid and digestion was verified by Eastern blot using *Maackia amurensis* lectin (MAL II) or Sambucus nigra lectin (SNA). (**d**) Monolayers of HaCaT cells were treated with AK23 lacking sialic acid (10 µg/mL). (**e**) AK23 were treated with galactosidase (+) or not (−) to remove galactose and digestion was verified by Eastern blot using *Erythrina cristagalli* lectin (ECA). (**f**) Monolayers of HaCaT cells were treated with AK23 lacking galactose (10 µg/mL). One experiment representative of three is shown. Patients’ IgG from active phase were treated (+) or not (−) with neuraminidase to remove sialic acid and digestion was verified by Eastern blot (patient 1 in (**g**), patient 2 in (**i**) and patient 3 in (**k**)). Monolayers of HaCaT cells were treated with purified IgG collected during the active phase lacking (right) or not (left) sialic acid (62.5 µg/mL) (patient 1 in (**h**), patient 2 in (**j**), patient 3 in (**l**)). Pictures were taken with EVOS XL core microscope magnification ×2.

The incubation of AK23 mAb on cell sheets of HaCaT cells resulted in the dissociation of the cell sheets in small fragments, while the cell sheets incubated with the control IgG from healthy donors (HD) exhibited minimal dissociation (Figure 1a). The typical *N*-glycan structure is shown in Figure 1b, with sialic acid that could be present, or not, on the terminal extremity of the *N*-glycan attached to the IgG. In order to evaluate the role of *N*-glycans in the pathogenic activity of autoantibodies, AK23 mAb was treated sequentially with neuraminidase, in order to remove sialic acids, and then by galactosidase to remove terminal galactose residues. Eastern blot analysis, using *Maackia amurensis* (MAL) or *Sambucus nigra* (SNA) lectin, and *Erythrina cristagalli* (ECA) lectins, showed complete digestion with neuraminidase (Figure 1c) and galactosidase (Figure 1e), respectively. The pathogenic activity of modified AK23 mAb was then measured in vitro on an HaCaT cell sheet. The AK23 mAb with no sialic acid dissociated the cell sheet 2.05 ± 0.63-fold less than the native AK23 mAb (*n* = 3) (Figure 1d), suggesting that the *N*-glycans present on AK23 mAb could modify its pathogenicity. In contrast, the AK23 mAb with no galactose dissociated the cell sheet more efficiently than the native AK23 mAb (Figure 1f). Purified IgGs, from patients with active pemphigus, were then treated by neuraminidase. Eastern blot analysis with MAL showed partial digestion, but the use of SNA lectin showed complete digestion in all three of the patients analyzed (Figure 1g,i,k). The sialic acid-depleted patients’ IgG dissociated the HaCaT cell sheet less than the patients’ native IgG (Figure 1h,j,l). Altogether, these results demonstrated that the *N*-glycans expressed by the patients’ IgG modified their pathogenic activity in vitro.

### 3.2. Pathogenic Activity of IgG Antibodies from Patients with Pemphigus

Pemphigus sera were collected from a randomized clinical trial, in which 45 patients were treated with corticosteroids. Of these, 11 patients still had detectable anti-DSG3 IgGs at the following time points analyzed: day 0, day 180, day 365, and day 730. Only 7 of these 11 sera were available in sufficient quantity for the experiments. Of the seven patients, two achieved complete remission during the course of the study, and we analyzed one of these two sera (patient 3) because it contained high titers of anti DSG3 Abs. The other five patients were in incomplete remission and relapsed several times. Similarly, we selected the two of these five sera that contained the highest titers of anti-DSG3 Abs (patients 1 and 2). Purified IgGs from three pemphigus patients were studied longitudinally, from day 0 to day 730. The main patients’ baseline characteristics are shown in Table 1. All the patients had a mucocutaneous PV. The evolution of corticosteroid doses and anti-DSG antibodies are shown in Supplemental Figure S1 and Figure S2, respectively.

**Figure 2:**
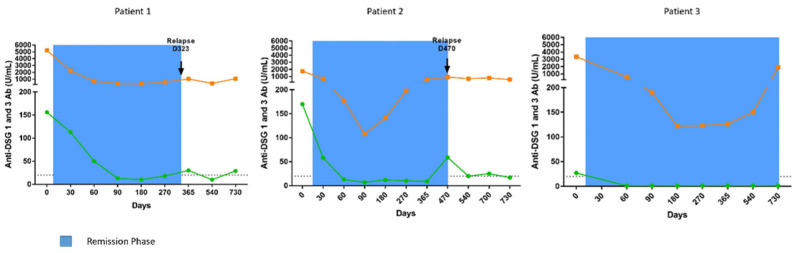
Evolution of anti-DSG1 (green) and anti-DSG3 (orange) antibody titers in sera from pemphigus patients treated with corticosteroids. Anti-DSG1 and anti-DSG3 IgG antibody titers were measured by ELISA. Dashed line represents the cut-off values proposed by the manufacturer for anti-DSG1 and anti-DSG3 antibody ELISA values. The remission phase is indicated in blue.

All three patients initially had a major drop in anti-DSG1 and anti-DSG3 antibody levels after the start of treatment, corresponding to between a 91.7% and 100% reduction, relative to the baseline, followed by the progressive re-increase in their anti-DSG3 antibodies before the occurrence of relapses from day 365 to day 730 (Figure 2). 

**Figure 3:**
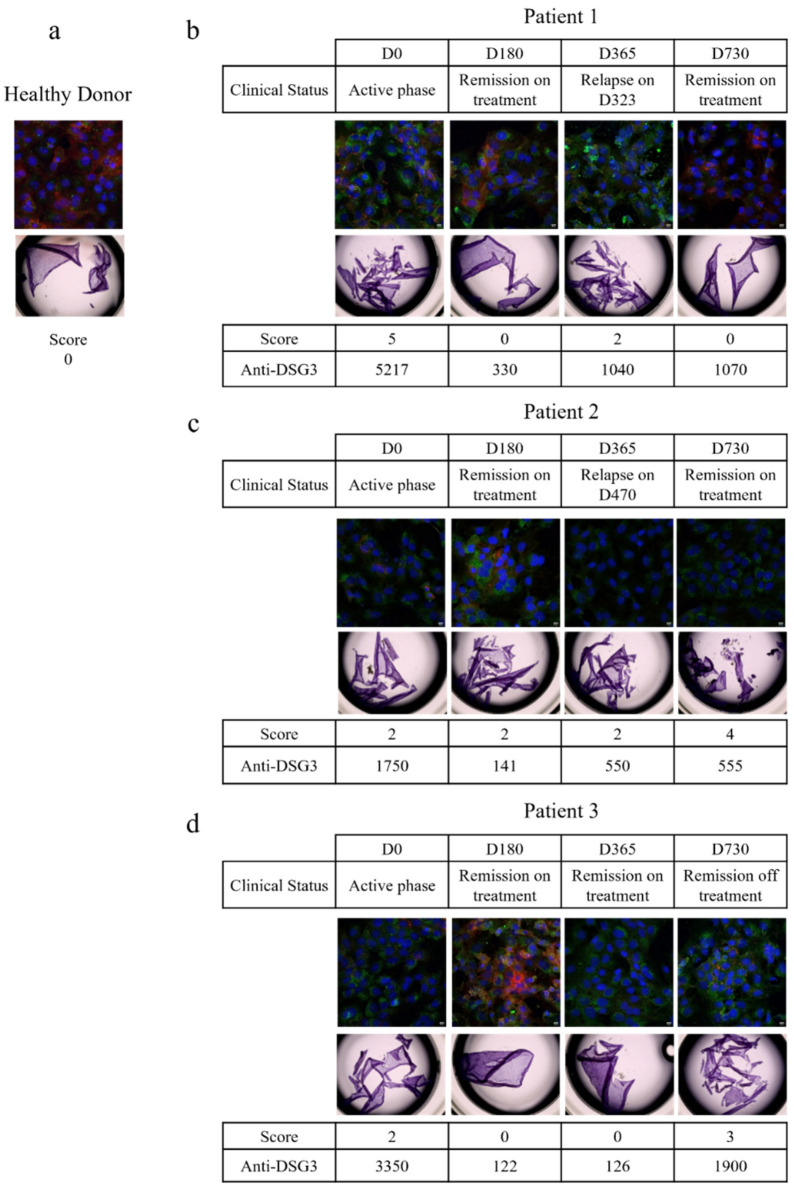
Longitudinal study of IgG pathogenicity. Pathogenicity of IgG from healthy donors (**a**) and three different pemphigus patients (**b**–**d**) were studied longitudinally by dispase-based dissociation assay and immunofluorescence. For the dispase-based assay, monolayers of HaCaT cells were treated with 62.5 µg/mL of purified IgG from healthy donors or pemphigus patients for 24 h. For immunofluorescence, cells were treated with 62.5 µg/mL of purified IgG from healthy donors or pemphigus patients for 20 h. Then cells were fixed and immuno-stained for DSG3 (red) and flotillin-2 (green). DAPI was used for nuclear staining (blue). Healthy donors: *n* = 5; patients: *n* = 3. Five images were taken for each condition, in two independent experiments. Pictures were taken with a Leica SP8-UV confocal microscope magnification ×40.

The in vitro pathogenic activity of purified serum IgG was then tested by the following two different in vitro methods: the dispase-based dissociation assay, as described above, and using an immunofluorescence assay on the HaCaT cell line. For the latter, the presence of autoantibodies leads to a loss in the detection of DSG3 and a redistribution of the cytoskeleton, as observed with the flotillin-2 staining. IgGs from five HD were used as the control (Figure 3a).

#### 3.2.1. Patient 1

The patients’ serum collected during the active phase of the disease (at day 0) induced a loss in DSG3 staining by immunofluorescence, a redistribution of flotillin-2, and fragmentation of the cell monolayer, indicating that the serum IgGs were pathogenic (Figure 3b). At day 180, when the patient was in clinical remission, with a 93.7% decrease in anti-DSG3 antibodies, his IgG did not induce fragmentation of the cell layer anymore, and a nice staining of the DSG3 was observed, while a slight redistribution of flotillin-2 was still observed, indicating that the patient’s IgGs had lost most of their pathogenic properties. The patient relapsed at day 365, and the immunofluorescence and cell dissociation assays performed at the time of relapse showed that the IgGs were pathogenic again. Finally, the patient’s serum IgGs that were collected at the latest evaluation at day 730, when the patient was in clinical remission again, did not show any pathogenic activity, while, surprisingly, the anti-DSG3 IgG ELISA values of the two sera collected at day 365 (relapse) and day 730 (remission) were very close (1040 vs. 1070 IU/mL). This patient further relapsed between day 730 and day 1080, corresponding to the 12-month evaluation after the end of the study. 

#### 3.2.2. Patient 2

The patient serum IgG collected at the baseline evaluation contained high titers of anti-DSG3 antibodies, and induced disruption of the DSG3 staining and cell layer fragmentation (Figure 3c). At the day 180 evaluation, the anti-DSG3 ELISA values dropped down from 1750 IU/mL at baseline to 141 IU/mL. A slight redistribution of flotillin-2 was still visible, while a certain reappearance of the DSG3 staining was observed, as compared with the baseline serum IgG sample. The patient further relapsed at day 470. The patient serum IgG was collected at day 730 and still had pathogenic activity, which was in accordance with the occurrence of a second relapse between day 730 and day 1080. 

#### 3.2.3. Patient 3

At baseline, the patient’s serum IgGs were pathogenic, as demonstrated by the loss of DSG3 staining, redistribution of flotillin-2, and fragmentation of the cell layer (Figure 3d). At day 180, when the patient was in clinical remission after treatment, the DSG3 staining reappeared and the cell layer was not fragmented anymore, indicating that the IgGs were no longer pathogenic. At days 365 and 730, while the patient was in sustained clinical remission, his serum IgG showed variable signs of in vitro pathogenic activity in the cell layer fragmentation test, and DSG3 and flotillin-2 immunofluorescence assays. This patient further relapsed at day 820.

Overall, these results showed a strong correlation between clinical activity, serum anti-DSG3 antibodies titers, and demonstration of the in vitro pathogenic activity of serum IgG at the onset of pemphigus, while the achievement of clinical remission under treatment, at month 6, was also strongly correlated with a drop in anti-DSG antibodies, and a loss of the pathogenic activity of patients’ IgG. Interestingly, a persistent pathogenicity of serum IgG was observed in patients 2 and 3, who were in clinical remission at the time that their sera were studied, but who further relapsed within a few months after the evaluation of their sera. Finally, no pathogenic activity was detected at the day 730 evaluation of the serum from patient 1, despite persistent high titers of anti-DSG3 antibodies. Unfortunately, no serum from this patient was collected during the period from the day 730 evaluation to the time of relapse, which did not allow us to assess the pathogenic activity of IgGs closer to the time of relapse.

### 3.3. IgG N-glycosylation Profiles

**Figure 4:**
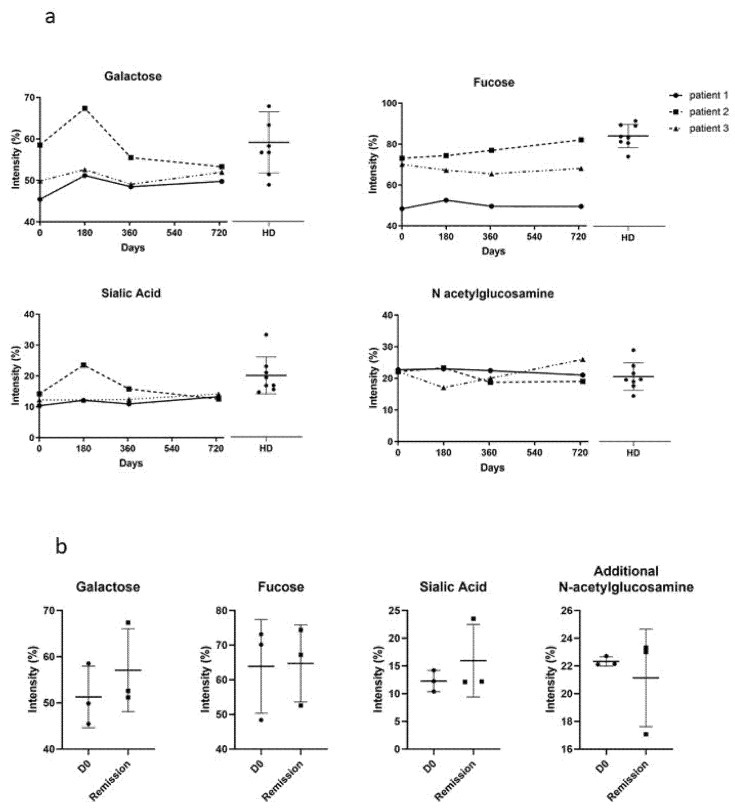
Study of the *N*-glycan profiles of IgG. (**a**) Comparison of the IgG galactosylation, fucosylation, sialylation and presence of additional *N*-acetylglucosamine in healthy donors (HD) and pemphigus patients in active phase (D0). (**b**) Comparison of the IgG galactosylation, fucosylation, sialylation and presence of additional *N*-acetylglucosamine in active (D0) and remission phase of pemphigus patients. HD: *n* = 8; patients: *n* = 3.

Because IgG *N*-glycosylation is altered in autoimmune and chronic inflammatory diseases, and modulates the immune response [33], we studied the *N*-glycosylation profiles of purified IgGs from eight HD and three PV patients, and analyzed their evolution during the course of the disease, at day 0, day 180, day 365, and day 730 (Figure 4a). The *N*-glycans on purified IgGs from HD and PV patients were enzymatically released, purified, and permethylated prior to MALDI-TOF MS analysis (Supplemental Figures S2 and S3). Particular attention was paid to the different *N*-glycan structures, including the ones bearing galactose, fucose, sialic acid, and additional *N*-acetylglucosamine residues. First, we longitudinally analyzed the proportion of galactose, fucose, sialic acid, and additional *N*-acetylglucosamine in all the patients. We showed that IgG *N*-glycans varied depending on the patient and their evolution, but that *N*-glycans were relatively stable over the course of pemphigus in each patient, and were in the range of the variation observed in HD (Figure 4a). Second, we compared the relative proportions of the different substitutions (galactose, fucose, sialic acid, and additional *N*-acetylglucosamine) between the samples collected in patients at day 0 (active disease) and at day 180 (clinical remission) (Figure 4b). No significant difference was observed for galactose, fucose, sialic acid, and additional *N*-acetylglucosamine (51 ± 7% vs. 57 ± 9%, *p* = 0.42; 64 ± 14% vs. 65 ± 11%, *p* = 0.93; 12 ± 2% vs. 16 ± 7%, *p* = 0.40; 22 ± 0% vs. 21 ± 4%, *p* = 0.60, respectively). Altogether, we did not find evidence for a correlation between the IgG *N*-glycans profile in the active phase and in the remission phase of pemphigus.

## 4. Discussion

In this study, we showed that (i) modifications of the *N*-glycan structure of patients’ IgG or the AK23 anti-DSG3 mAb modified its in vitro pathogenicity; (ii) IgG *N*-glycans profiles seemed to be relatively stable during the course of pemphigus, and did not seem to be correlated with the in vitro pathogenic effects.

Antibody Fc *N*-glycosylation can modify the function of antibodies by determining which Fc receptors they can bind to, in order to recruit effector cells. Modifications in *N*-glycosylation are thus a critical mechanism for improving the therapeutic efficacy of antibodies, by increasing their effector function, as demonstrated by the increase in the antibody-dependent cellular cytotoxicity (ADCC) of non-fucosylated rituximab [34]. Distinct vaccine regimens have been demonstrated to induce different antigen-specific IgG *N*-glycosylation profiles, suggesting that antibody *N*-glycosylation can be modified by various inflammatory signals during B cell priming [35,36]. A shift in patients’ antibodies to a highly inflammatory *N*-glycosylation profile, characterized by galactosylation, has been shown in various autoimmune diseases, such as RA, SLE, and autoimmune vasculitis [20,22,37,38], indicating a pathogenic effect of low galactose levels.

In the present study, we did not observe any major difference in the galactosylation level between patients’ IgG and HD IgG, as well for sialic acid, fucose, and *N*-acetylglucosamine, for the duration of the study (Figure 4a). However, a low level of IgG sialylation has been reported in patients with RA and various autoimmune diseases [37,38,39,40,41,42], while IgG sialic acid has an anti-inflammatory role in RA [37,38]. Unfortunately, the evolution of these modifications of IgG sialic acid in patients with RA have not been studied after treatment. Similarly, the *N*-glycosylation profile of IgG in other autoimmune diseases has only been studied in patients with active diseases, but not after the start of treatment. Such a longitudinal analysis of the serum IgG *N*-glycome over several years has been reported in HD. It showed that despite the fact that high inter-individual variability was observed, only small changes could be detected in a single person [39]. These findings suggest that the few changes we observed at different time points, in the three patients of our study, were likely related to their clinical status under treatment. 

We showed that the in vitro pathogenic activity of AK23 mAb and that of the patients’ serum IgG were reduced by sialic acid removal, which might appear to be somewhat contradictory to the decreased usage of sialic acid in patients with active autoimmune disease (Figure 1). This discrepancy might be explained by a differential effect in vivo and in vitro. Indeed, sialylated IgGs have a lower affinity to the Fc receptors that reduce their ability to induce ADCC. Terminal sialylation confers anti-inflammatory effects in vivo [40], while, in vitro, anti-DSG3 antibodies can directly interfere with cell adhesion by steric hindrance of DSG adhesion or its internalization [6]. Interestingly, AK23 mAb has a putative *N*-glycosylation site in its CDR2, which could affect its affinity to DSG3 following neuraminidase digestion. In addition, the treatment of AK23 with galactosidase restored its pathogenic activity, indicating that the pathogenic activity of AK23 can be modified differently according to the various substitutions in its *N*-glycans. This highly suggests that modifications of the *N*-glycosylation of AK23 may affect its affinity for DSG3, either directly, by modifying the antigen binding site, or indirectly, by modifying the antibody structure [16,43,44,45].

We then studied a potential relationship between the in vitro pathogenic activity of the patients’ IgG, their *N*-glycan profiles, and pemphigus clinical activity. During the acute phase of the disease, clinical severity was well correlated with high titers of anti-DSG3 antibodies, and a strong in vitro pathogenic activity of patients’ serum IgG (Figure 3). At day 180, at the end of the initial treatment phase, a strong correlation was also observed between the achievement of clinical remission, low titers of anti-DSG3 antibodies, and the decrease or loss of the pathogenicity of serum IgG (Figure 3). Interestingly, pathogenic activity was detected in two of three sera (patients 2 and 3), while these patients were still in clinical remission, corresponding to a few months before the time at which a relapse occurred. No pathogenic activity was detected at the day 730 evaluation in the serum from patient 1, despite the fact that this serum contained high levels of anti-DSG3 antibodies. Unfortunately, no serum was collected from this patient from the last evaluation at day 730 to the time at which the patient relapsed (Figure 3). There were no differences in the IgG *N*-glycans profiles between the three patients, which allows us to associate the pathogenic activity of purified IgG to a particular *N*-glycans profile (Figure 4a).

Apart from the well-documented changes in IgG Fc N-glycosylation during inflammation, N-glycans attached to Fab might also have a role in modulating autoantibody activity [18]. Putative N-glycosylation sites in the Fab might not always become glycosylated, but Fab N-glycosylation has been found to either enhance or prevent antigen binding [43]. In mice, it has been shown that Fab N-glycosylation can prevent potentially autoreactive antibodies from becoming autoreactive, by lowering their affinity for self-antigens [46]. However, in patients with RA, the presence of such changes has not been shown to correlate with disease development or the resolution of inflammation [47].

In this study, the changes observed in the activity of AK23 mAb on the keratinocyte cell line were strikingly dependent upon whether the sialic acids or the galactoses were removed. We hypothesize that these observations, in the absence of complement or any Fc receptor-expressing cells, might be related to Fab glycosylation. It would have been of great interest to be able to purify anti-DSG3 antibodies from the patients’ sera, in order to compare N-glycans associated with the Fc domain or the Fab. Unfortunately, the quantity of N-glycans from the anti-DSG3 IgGs recovered from the DSG3 affinity column was not sufficient to obtain an interpretable mass spectrum. The limited quantity of serum available, and the sensitivity of the technic used, did not allow us to successfully perform these experiments on purified anti-DSG autoantibodies.

One limitation of our study is that the assays used to measure the pathogenic activity of autoantibodies were performed in vitro, in the absence of complement and Fc receptor-expressing cells, whereas altered *N*-glycosylation can modify the complement pathway [13] or the binding to the Fc receptor [17]. One may hypothesize that the use of an in vivo passive transfer neonatal mouse model might have provided different results regarding the relatively stable *N*-glycan profile during the evolution of patients that we evidenced in the present study.

Overall, this study showed that the pathogenic activity of serum IgG from patients with pemphigus was closely correlated with the evolution of clinical lesions during the initial phase of treatment. Thereafter, the persistence or reappearance of the pathogenic activity of serum IgGs in patients in clinical remission seems to be predictive of relapse. The *N*-glycan profile of pemphigus IgG is different from that of HD, but seems to be relatively stable during the evolution, although inter-individual variations could be observed.

## Figures and Tables

**Table 1:** Baseline characteristics of patients with pemphigus treated with corticosteroids.

Patients	3
Age, mean	69.4
Sex	
Female	2
Male	1
BMI, mean	24.46
Type of pemphigus	
Vulgaris	3
Foliaceus	0
Initial Presentation	
Mucosal	0
Cutaneous	0
Mucocutaneous	3
PDAI score, mean	45.6
Treatment	corticosteroids

## Data Availability

All data are available upon request.

## Supplementary Materials

The following are available online at https://www.mdpi.com/article/10.3390/biomedicines9101411/s1. Figure S1. Evolution of prednisone doses (mg/day), from D0 to D730. Plain line with circle = patient 1; dashed line with square = patient 2; dotted line with triangle = patient 3. Figure S2. Example of a mass spectrum of IgG Fc *N*-glycan from healthy donor. Blue square, *N*-acetylglucosamine; red triangle, fucose; green circle, mannose; yellow circle, galactose; purple diamond, *N*-acetylneuraminic acid. Glycans are drawn according to the international nomenclature recently updated [32]. Figure S3. Example of mass spectrum obtained for IgG Fc *N*-glycans from a pemphigus patient at D0. Blue square, *N*-acetylglucosamine; red triangle, fucose; green circle, mannose; yellow circle, galactose; purple diamond, N-acetylneuraminic acid that belongs to the sialic acids. Glycans are drawn according to the international nomenclature recently updated [32].
