# Supplementary material for: Longitudinal Pathogenic Properties and N-Glycosylation Profile of Antibodies from Patients with Pemphigus after Corticosteroid Treatment
Source: Biomedicines. 2021 Oct 8;9(10):1411. doi: 10.3390/biomedicines9101411 (PMC8533488; doi:10.3390/biomedicines9101411)
Supplement: Supplementary file 1 [file biomedicines-09-01411-s001.zip › biomedicines-1351953-supplementary.pdf]

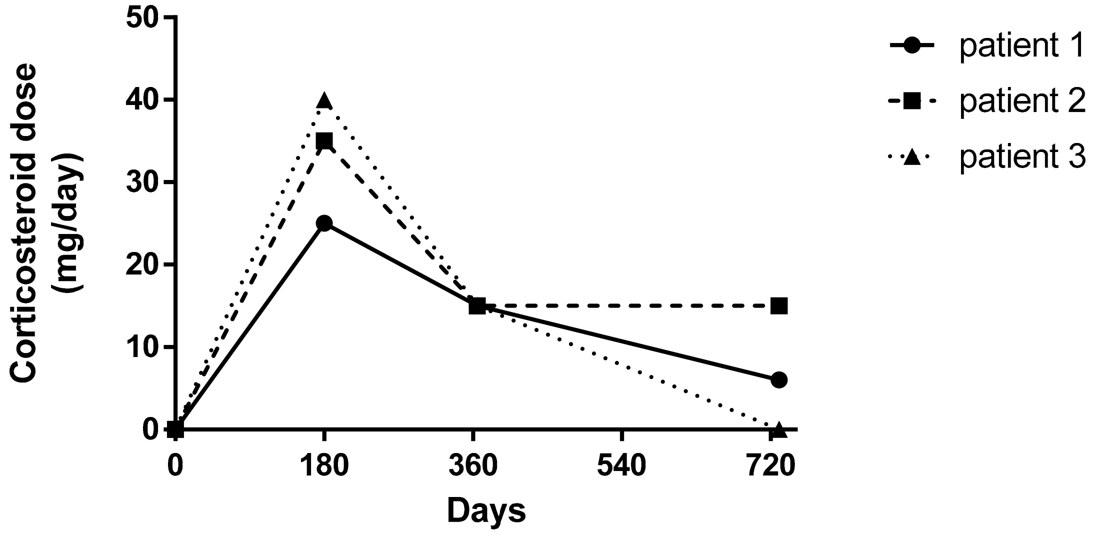

Supplemental Figure S1. Evolution of prednisone doses (mg/day), from D0 to D730. Plain line with circle = patient 1; dashed line with square = patient 2; dotted line with triangle = patient 3.

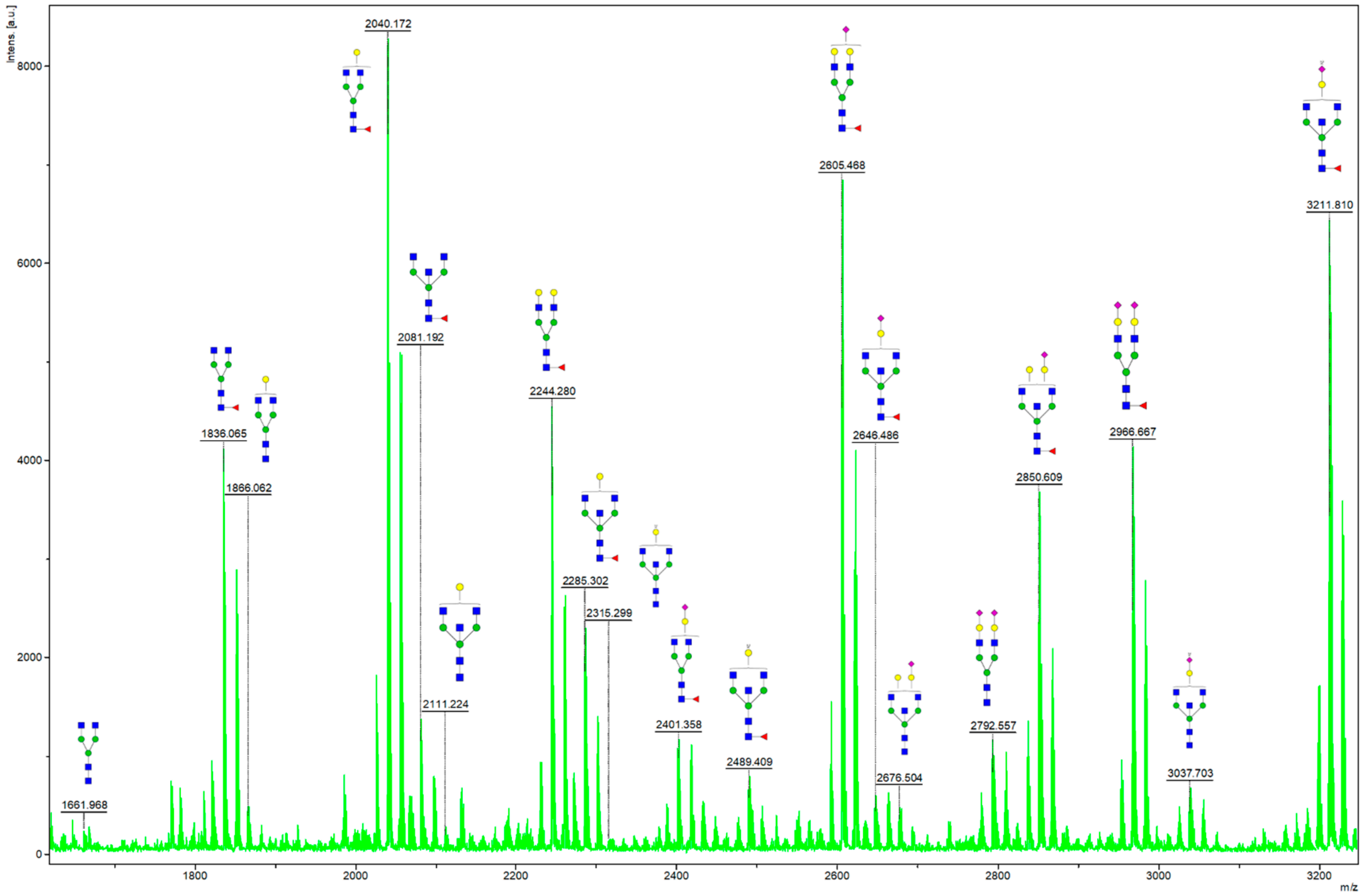

Supplemental Figure S2. Example of a mass spectrum of IgG Fc *N*-glycan from healthy donor. Blue square, *N*-acetylglucosamine; red triangle, fucose; green circle, mannose; yellow circle, galactose; purple diamond, *N*-acetylneuraminic acid. Glycans are drawn according to the international nomenclature recently updated [32].

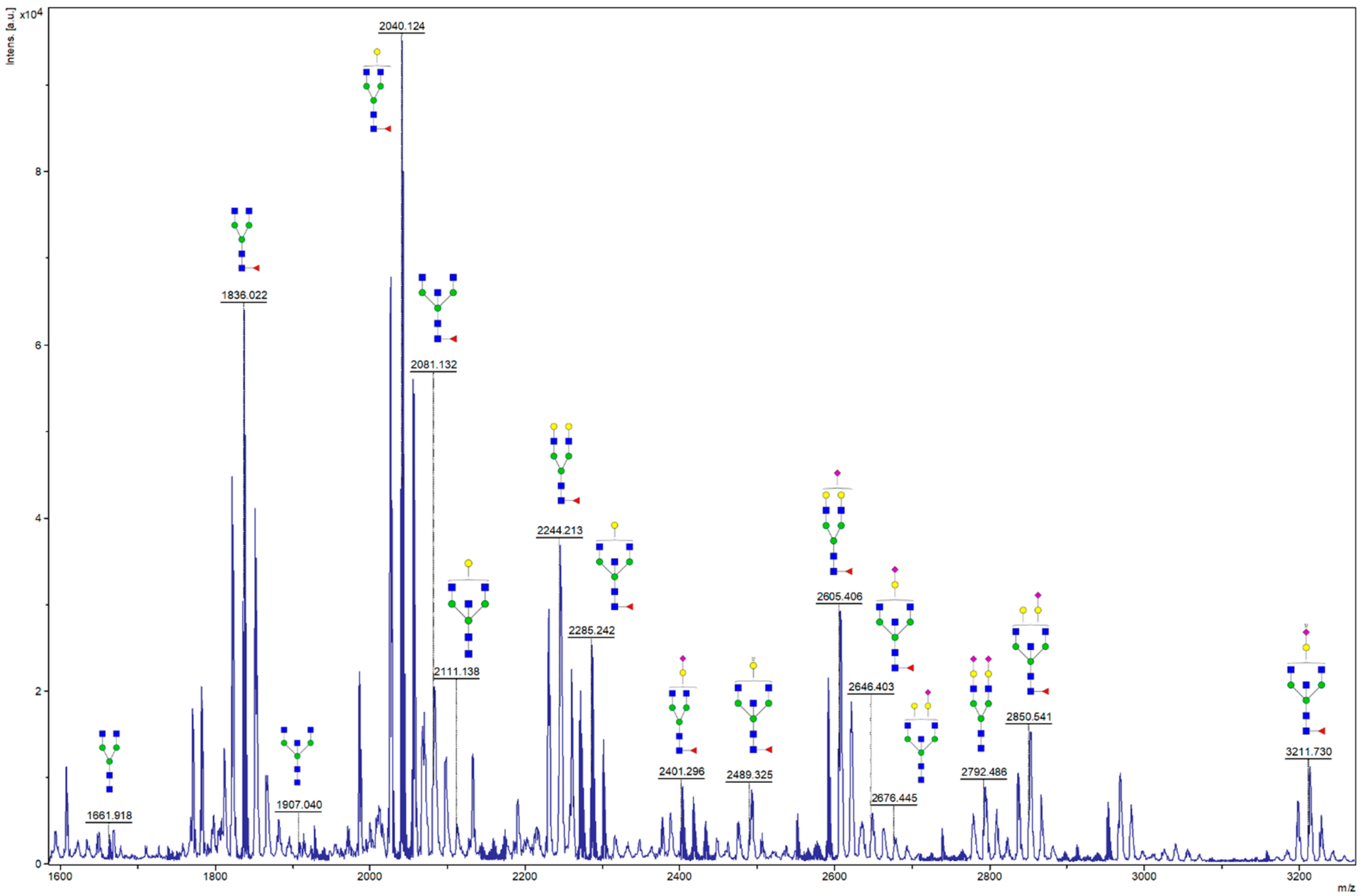

Supplemental Figure S3. Example of mass spectrum obtained for IgG Fc *N*-glycans from a pemphigus patient at D0. Blue square, *N*-acetylglucosamine; red triangle, fucose; green circle, mannose; yellow circle, galactose; purple diamond, N-acetylneuraminic acid that belongs to the sialic acids. Glycans are drawn according to the international nomenclature recently updated [32].
